# The Management of Iron Chelation Therapy: Preliminary Data from a National Registry of Thalassaemic Patients

**DOI:** 10.1155/2011/435683

**Published:** 2011-06-05

**Authors:** Adriana Ceci, Laura Mangiarini, Mariagrazia Felisi, Franco Bartoloni, Angela Ciancio, Marcello Capra, Domenico D'Ascola, Paolo Cianciulli, Aldo Filosa

**Affiliations:** ^1^Consorzio per Valutazioni Biologiche e Farmacologiche, Via L. Porta 14, 27100 Pavia, Italy; ^2^I.RI.D.I.A. srl, Viale Concilio Vaticano II 75/B, 70124 Bari, Italy; ^3^U.S. Dipartimentale Ematologia-DH Talassemia, Ospedale “Madonna delle Grazie”, Via Montescagliaso C. da Cattedra Ambulante 1, 75100 Matera, Italy; ^4^UOC Ematologia-Emoglobinopatie, Ospedale “G. Di Cristina”, Via Benedettini 1, 90100 Palermo, Italy; ^5^Centro Microcitemia, Azienda Ospedaliera “Bianchi Melacrino Morelli”, Via Melacrino 1, 89126 Reggio Calabria, Italy; ^6^U.O. Day Hospital Talassemia, Ospedale “S. Eugenio”, Piazzale Umanesimo 10, 00144 Roma, Italy; ^7^UOS Talassemia Pediatrica ed Emoglobinopatie Pediatriche, Azienda Ospedaliera di Rilievo Nazionale “Antonio Cardarelli”, Via A. Cardarelli 9, 80131 Napoli, Italy

## Abstract

Thalassaemia and other haemoglobinopathies constitute an important health problem in Mediterranean countries, placing a tremendous emotional, psychological, and economic burden on their National Health systems. The development of new chelators in the most recent years had a major impact on the treatment of thalassaemia and on the quality of life of thalassaemic patients. A new initiative was promoted by the Italian Ministry of Health, establishing a Registry for thalassaemic patients to serve as a tool for the development of cost-effective diagnostic and therapeutic approaches and for the definition of guidelines supporting the most appropriate management of the iron-chelating therapy and a correct use of the available iron-chelating agents. This study represents the analysis of the preliminary data collected for the evaluation of current status of the iron chelation practice in the Italian thalassaemic population and describes how therapeutic interventions can widely differ in the different patients' age groups.

## 1. Introduction

Thalassaemia major is a chronic, progressive haemoglobin disorder requiring life-long blood transfusions. However, in absence of an appropriate treatment, iron overload is the clinical consequence of chronic transfusions that can cause significant organ damage, morbidity, and mortality. Historical data show that iron overload in transfusion-dependent patients not only is fatal, but causes early death usually associated with cardiac complications [1]. 

Iron chelation treatment is necessary for the removal of excess iron, but treatment's efficacy and success are highly dependent on type and timing of the therapy as well as on patient adherence to treatment [2–5]. 

Only one chelating agent, deferoxamine (DFO), was available for many years. DFO was approved for clinical use in patients with iron overload due to frequent blood transfusions (including thalassaemia and other congenital anaemia), and to date, according to the existing national or international guidelines [6–8], it still represents the standard iron-chelating therapy. In the last 50 years, deferoxamine has proven to be safe and efficacious, radically changing the prognosis of transfusion-dependent patients. However, despite this benign safety and efficacy profile, the parental administration, with the nuisance of an infusion pump (for overnight subcutaneous infusion 5 to 7 nights/week), highly prevents optimal compliance, particularly among the youngest patients [9–13]. Moreover, almost 10–15% of subjects are unable to use this chelator due to hypersensitivity or toxic side effects and the majority of patients refuse DFO because of the poor quality of life associated with the treatment.

These difficulties prompted in recent years the search of an orally administrable compound. Research led to the identification of several interesting molecules but, among these, only two agents became available on the European market: deferiprone and deferasirox. 

Deferiprone (DFP) was approved in 1999 and for many years was the only oral chelator available in Europe for the treatment of patients for whom deferoxamine was contraindicated or presented serious toxicity. Its use determined significant improvements in terms of compliance and quality of life [14–16]. In addition, there are clinical evidences that DFP is more effective in the protection against iron cardiotoxicity than DFO, thus increasing patient's survival and reducing significant morbidity [17, 18]. 

These findings also stimulated the combined use of the two drugs with the aim to obtain a global improvement and reduced toxicity in treating iron overload. In particular, the combination therapy originates from the observation that patients have variable responses in iron excretion and toxicity with these two chelating agents, and their combination could increase the overall iron excretion and reduce the toxicity. It is hypothesised that deferiprone, able to cross cell membrane, and thus reaching pools of iron not accessible to DFO, could shuttle tissue iron to deferoxamine in the blood stream, where it can be chelated and eventually excreted [19, 20].

In Italy, deferiprone was introduced in 1997 within a National Controlled Program prior to the European marketing authorisation approval. At that time, 532 patients from 86 clinical centres were prescribed the new oral-chelating treatment. The Program included paediatric patients (10.9% were 6–11 years of age) but no children below 6 years of age were enrolled [21].

During the years DFP, either alone or in combination with DFO, became the preferred treatment for older children and adults in need to reduce cardiac iron overload. In 2010, on the basis of evidence from sponsored and spontaneous clinical trials, the European Medicinal Agency (EMA) updated the registrative dossier reporting that “Data from the published literature are consistent with the result from the Apotex studies, demonstrating less heart disease and/or increased survival in Ferriprox-treated patients than in those with deferoxamine” [22]. 

Recently a new oral chelator, deferasirox (DFX), has been approved in Europe for adults and children above 6 years when transfused with 7 mL/Kg/die or more. The product is also approved as a second line for children 2–6 years with beta-thalassaemia and transfused with less than 7 mL/Kg/die or with other transfusion-dependent anaemia. Its long half life (11–19 hours) allows maintaining plasma levels within the therapeutic range for 24-hour period, permitting a convenient once-daily oral administration. Long-term data have not shown any increase or unexpected adverse event with deferasirox therapy in both paediatric and adult patients, although some concerns have recently been raised for some evidence of cytopenia and renal toxicity [23]. DFX seems to be the preferred chelator in terms of compliance and quality of life, even if the unpleasant taste and the high rate of not serious adverse reactions of gastrointestinal nature reduce the acceptability and the generally expected advantages. 

The availability of alternative therapies, with differentiated mechanism of action and tissue sensitivity, could play a crucial role in the continuous improvement of the thalassaemic patient's survival and quality of life. However, consensus guidelines on the optimal use of the available chelators are still in preparation. 

It would be of great interest to identify the characteristic of patient populations which can best benefit from the use of each chelator. In lack of prospective controlled comparative trials, we can derive useful information from the analysis of the current use of the different chelators and of the medical decision underlying the prescription. The critical evaluation of such current uses, in view of the existing guidelines and regulatory provisions, should encourage a rationale use of these life-saving drugs. 

### 1.1. Aim of the Study

In Italy, the “Inter-regional Network for Thalassaemia: HTA for the diagnostic and therapeutic intervention for iron overload” is a project recently promoted and founded by the Italian Minister of Health. Within the framework of this project, a registry of thalassaemia patients was designed and set up to provide a flexible platform for the assessment of the Thalassaemia major patient's characteristics and disease management, including the utilization of the different chelating agents. Other information collected include the outcome of the treatment, the adverse events rate, the methodologies for iron deposition evaluation, and the cost of therapies. The project is still ongoing and the final results will be made public.

The present study represents a preliminary report of the first set of data collected in the registry. Data have been used to perform a prevalence study aimed at evaluating

- the demographic characteristics of the study population 11 and 5 years after the introduction of the two oral chelators DFP and DFX, respectively,
- the current management of iron overload in a large cohort of patients of different age subsets.

## 2. Materials and Methods

### 2.1. Study Design and Sites

This is a multicenter, cross-sectional study on iron chelation treatment.

### 2.2. Study Population

The study population consists of subjects with confirmed diagnosis of beta-thalassaemia major and under any chelation treatment in the 15 participating Clinical Centres:

- Ospedale “Madonna delle Grazie” U.S. Dipartimentale Ematologia-DH talassemia (Dr A. Ciancio),
- A.O. Bianchi Melacrino Morelli Centro Microcitemia (Dr. D. D'Ascola),
- Ospedale “Cardarelli” UOS Talassemia Pediatrica ed Emoglobinopatie Pediatriche (Dr. A. Filosa),
- A.O.R.N. “Cardarelli” U.O.C. Microcitemia (Dr. L. Prossomariti),
- Osp. “S. Eugenio” U.O. Day hospital Talassemia (Dr. P. Cianciulli),
- Unità operativa complessa di genetica ed immunologia pediatrica—Servizio di Microcitemia Azienda Ospedaliera Universitaria “G. Martino” (Dr. B. Piraino),
- Presidio ospedaliero Centro Immunotrasfusionale (Dr. A. Di Caro),
- Centro di riferimento regionale per la diagnosi e cura delle emoglobinopatie. A.O.V. Cervello (Dr. G. Calvaruso),
- U.O.C. Ematologia—Emoglobinopatie, Ospedale G. Di Cristina, Azienda di Rilievo nazionale e di alta specializzazione Civico, Di Cristina, Ascoli—Palermo (Z. Borsellino),
- Ospedale “Villa Sofia” U.O.S di talassemia (Dr. L. Pitrolo),
- P.O. “S. Bambino” U.O.C. Servizio di Talassemia (Dr. G. Colletta),
- Talassemia Azienda O.U. Policlinico (Dr. M. A. Romeo),
- Azienda Ospedaliera O.C.R. SCIACCA U.O.S. di Talassemia (Dr. C. Gerardi),
- A.O. Umberto I U.O.S. Talassemia (Dr. S. Campisi),
- P.O.S. Luigi—S.Curro' U.O.D. Talassemia (Dr. S. Anastasi).

As the project is an initiative promoted by Basilicata, regions which were the first to adhere to the Registry were mainly from the south of Italy. For the aim of this study, data are collected from these first participant centres. To date, other haematological clinical centres from all over Italy adhered to the project and data collection is currently ongoing. 

Patients who underwent bone marrow transplantation or taking part in interventional studies have been excluded from the present study.

### 2.3. Data Collected

For each patient, the following data have been collected and analysed: (a) demographic characteristics (age and sex), (b) anamnesis information, (c) data on chelation therapy.

### 2.4. Guidelines and Approved Indications

Details on the recommended and authorised uses of the three different chelators are derived by the relevant existing Guidelines (Guidelines for the clinical management of thalassaemia. Thalassaemia International Federation—2008 [2]; Practice guidelines for the management of iron overload in thalassaemia major and related disorders. Italian Society of Hematology—2008 [3]; Consensus view on choice or iron chelation therapy in transfusional iron overload for inherited anaemia, UK Forum on Haemoglobin Disorders—2008 [4]) and from the approved Summary of Product Characteristics (SmPC) available in Italy for the three drugs.

### 2.5. Data Analysis

A descriptive analysis has been carried out by age, gender, iron chelation agent, and referring centre using proportions for categorical data, mean and median as central tendency parameters for continuous data, Standard Deviation (SD), and minimum (min) and maximum (max) values as dispersion parameters. 

Data were collected in compliance with the GCP procedures (Italian D.M. 15/07/97), the Guidelines for the classification and conduction of the observational clinical studies (Italian G.U. n.76, 31/3/2008), and the Guideline for the protection and treatment of personal data (Italian DL 196/2003).

## 3. Results and Discussion

### 3.1. Demographic Information

As in October 2010, a total of 981 subjects were registered in the 15 Italian Clinical Centres participating to the Registry for Thalassaemic patients, the majority of which were located in the south of Italy (Table 1). 

The smallest centre had in care 10 patients and the largest as many as 149 patients. The majority had in care both paediatric and adult patients, with 4 centres having only adult thalassaemic patients. 

Demographic characteristics of the patient population included in this study are summarised in Table 2. In the specific, gender distribution is balanced among males (47.2%) and females (52.8%). The global mean age (±SD) is 30.56 ± 10.47 years, with the youngest patient aged 2 years and the oldest patient being 60 years old. The paediatric population represents 11.5% of the study population (113 subjects are aged 2–18 years) and 95% of the patients are 45 years of age or younger.

**Figure 1:**
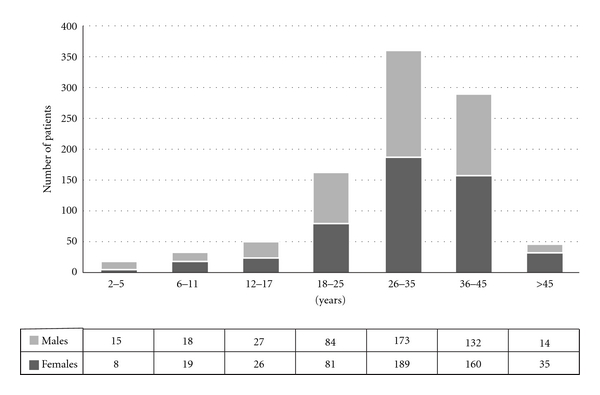
Patient distribution stratified by age and gender.

Details on the age distribution of patients receiving iron-chelating therapy are illustrated in Figure 1. 

On the basis of these figures, we can derive two main considerations. First of all it is noticeable that, despite the numerous efforts made in terms of prevention during the years, 6% of the thalassemic population in Italy are currently below 12 years of age and 11,5%, of patients are in the paediatric subset. The second observation concerns the mean age of the patient population which is increased to 30.56 ± 10.47. It was reported that the mean age of patients shifted from 5 years of age in 1965 to 27 years of age in 1995 [4]. As DFX has been introduced in Italy only recently, this global increase of the mean age can be attributed to the use of DFO and DFP.

### 3.2. Current Management of Iron Overload

When the patient population included in this study (981 patients) was analysed in terms of undergoing therapy, 495 patients resulted to be under treatment with DFO (as monotherapy or combined therapy). In detail, 271 patients (27,6% of the total) were receiving DFO, 185 (18,9%) were treated with DFP, and 304 subjects (31,0%) were receiving DFX (Table 3). The combination therapy with two iron-chelating agents was administered to 221 patients (22,5%), almost exclusively as a combination of deferoxamine and deferiprone both used in association (12,7%) or as sequential therapy (9,5%). Only 3 (0,3%) patients in our cohort were reported to be receiving deferoxamine and deferasirox in sequential therapy.

The analysis by centre shows a great variability in the prescriptive approach of the chelation therapy among the different clinical centres. The prescription rate of DFO can vary from 8.1% to 70.8% of patients, while the prescription rate of DFP monotherapy ranges from 3,8% to 49.5%, depending on the referring centre. DFX, the last approved drug, is almost exclusively used as monotherapy. Only DFO + DFP combined therapy appears to be more consistently prescribed. 

**Figure 2:**
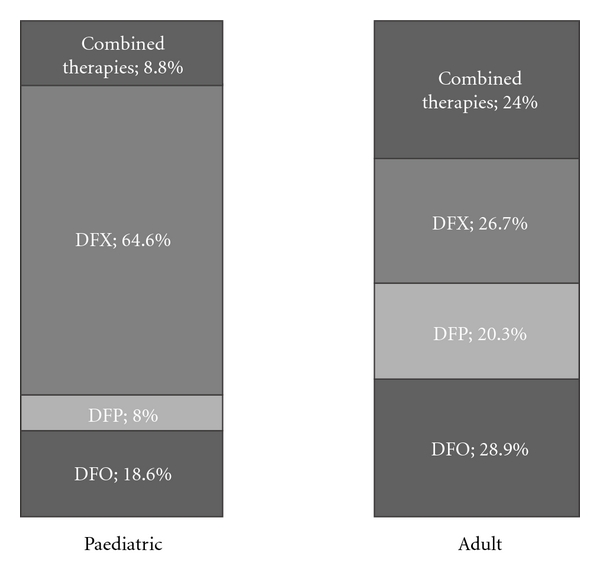
Iron chelation treatment in the paediatric and adult population.

The distribution of different chelating agents by age is illustrated in Figure 2. Adult patients appear to be equally distributed among the four available therapeutic choices (three chelators in monotherapy plus the combined treatment). On the contrary, in the paediatric population, there is a clear preference for prescribing an oral chelator (72,6%) and deferasirox is by large the preferred chelator in this age group (64.6%). 

These data indicate that many differences in terms of prescription rate of each iron chelator exist in the different age groups. These differences can be explained only partially on the basis of the approved products labels or of the existing clinical recommendations as detailed in Table 4. 

In this regard, preliminary results from a structured interview we are conducting with the participating clinical centres which aimed to explore the medical reasons for changing the prescribed iron chelation therapy, demonstrate that the main reason for switching from DFO to an oral iron chelator is almost exclusively the poor compliance associated to the parental drug in younger patients. On the other hand, every interviewed investigator considers cardiac iron overload the reason for initiating the simultaneous combination therapy (“Inter-regional Network for Thalassaemia” project, Internal Meeting, Rome, May 2010). This prescriptive approach confirms that doctors assign to deferiprone (mainly used in association with DFO) an important role as iron chelator particularly in the case of cardiac risk due to iron cardiac overload.

**Figure 3:**
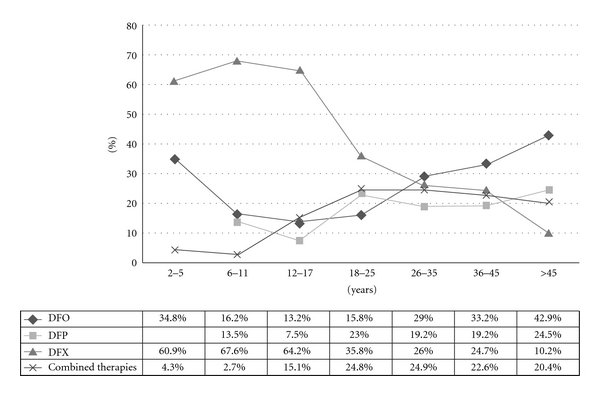
Iron-chelating treatment stratified by age group.

Figure 3 elucidates the variations among the different chelating treatments in the different age groups. 

In particular, the comparison of the prescription rate derived by the present study with the recommended uses in reported in the SmPC and guidelines highlights the following.

**2–5 Years**

No more than 35% of the small group of children below 6 years of age is currently under DFO monotherapy, the only iron-chelating agent authorized as a first line therapy in this group of age. Over 60% of them are using an oral chelator treatment (DFX) and, as this use is not authorized or recommended, it should be considered as an off-label use.

In addition, the use of DFO in combination with DFP in this age subset is very rare. As no major cardiac iron overload is expected in this age group, monotherapy should represent the easier and most appropriate treatment. Unfortunately, so far the only monotherapy which demonstrated efficacy and safety in this group of age is DFO for which, however, nightly subcutaneous route of administration remains an issue for youngest patients. The poor compliance to DFO in this age group is a problem which claims for conducting adequate oral chelator studies in younger children.

**6–11 Years**

Monotherapy with an oral chelator is the most largely used therapeutic option in this age group, with as many as 13.5% of patients using DFP and 67.6% using DFX (Figure 3). The prescription rates in this group demonstrate the rapid widespread among the youngest thalassaemic patients of the newly marketed oral-chelating agent DFX (authorised in Europe in 2006), favoured mainly by the convenient once-a-day administration, which allows overcoming the compliance problems associated with DFO, and positively compares to the three-time-a-day administration for DFP.

**12–17 Years**

The chelating therapy in this age subset follows a similar trend as above. The percentage of adolescents receiving deferoxamine is as low as 13% and the oral chelators are used in over 70% of teenagers and adolescents. Combined DFO + DFP treatment in the adolescent group strongly increases. Simultaneous administration accounts for 11.3% of the treatment in this age group and sequential therapy for 3.8%. This data is in line with the recognised need to start a robust iron-chelating programme in this age group in order to avoid cardiac impairment or, in the worse cases, to correct an overt cardiac iron overload. However, it should be noted that the combined use (both simultaneous and sequential) is not yet approved and spontaneous data available on safety/efficacy are to be confirmed with well-designed large controlled studies.

**18–45 Years**

The rate of patients treated with DFO (either used alone or as combination therapy with DFP) increases with the age and accounts for 30–40% of patients above 26 years of age.

It is interesting to note that the oral-chelating practice is radically different in older patients compared to the youngest. Indeed, the use of DFP (either alone or in combination with DFO) represents the current therapy for 40% of transfusion-dependent thalassaemic patients above 18 years of age, while DFX is used in only 25% of patients. The combined administration of two chelators (DFO + DFP) represents the therapy of choice in almost 25% as in the adolescent group. 

Finally, in our cohort two patients above 30 years of age were reported to be treated with the DFO/DFX combined therapy (sequential modality). However, this use is not authorised, and appropriate studies are warranted to evaluate safety, efficacy, and benefit/risk.

**>45 Years**

The use of DFO (alone or in combination) still remains the therapy of choice for thalassaemic patients above 45 years of age, followed by DFP that is used in 24.5% of patients. On the contrary, the use of DFX as monotherapy becomes as low as 10%.

## 4. Conclusions

This study highlights how DFO is excluded from the treatment of younger children and how oral chelators are preferred on the basis of the expected compliance advantage, despite the lack of marketing authorization approval. This compliance benefit is less recognized in the case of DFP that is often refused for safety reasons. However, EMA has updated the DFP SmPC, clearly indicating a potential superiority in cardiac iron chelation, as suggested also in the current guidelines, and it will be of interest to evaluate in future studies if this recommendation will be applied to the clinical practice. 

Concluding, in the recent years the main changes in prescription habits concern the dramatic increase in the use of the newly approved oral chelator DFX; this is particularly frequent not only among previously unchelated patients (from 2 years of age onwards), but also in older children and adults in substitution of DFO. 

In addition, this study highlights how many patients are still prescribed off-label. This is the case for the use of the oral chelator DFX in the 2–6 years age group (2,3% of the total population and 20,3% of the paediatric population) and for all the uses of the combined DFO+ DFP.

Finally, it is interesting to note that patients aged 45 and above appear reluctant to modify their current therapy, mainly consisting in DFO or DFP either as monotherapy or in combination. This evidence brings two significant implications for the patient's prognosis and safety. On one hand, the use of two chelators with slightly different mechanism of action has prolonged significantly the overall survival of the thalassaemic patients. The second observation is that, when a-well controlled chelation is achieved, the problem of compliance (which is almost complete in the adult population) becomes negligible and the maintenance of well being becomes the first objective for both patients and prescribers.

## Figures and Tables

**Table 1:** Thalassaemia clinical centres adhering to the study.

Region	Number of clinical centres	Number of patients
Sicilia	9	636 (64.8%)
Campania	2	149 (15.2%)
Lazio	1	83 (8.5%)
Calabria	1	53 (5.4%)
Basilicata	1	50 (5.1%)
Puglia	1	10 (1.0%)
Total	15	981 (100.0%)

**Table 2:** Demographic data of the patients participating to the study.

	Females	Males	Global
Sex (%)	518 (52.8%)	463 (47.2%)	981
Age: mean ± SD	31.45 ± 10.45	29.61 ± 10.37	30.56 ± 10.47
Median (range)	33 (2–60)	31 (2–54)	32 (2–60)
Paediatric patients (%)	53 (46.9%)	60 (53.1%)	113

**Table 3:** Percentage of use of the different chelating agents in the centres participating to the study and having in care both paediatric and adult patients.

Iron chelation treatment	Median (%)	Min–Max (%)
DFO	24.5	8.1–70.8
DFP	14.9	3.8–49.5
DFX	28.9	17.0–41.5
Combined therapy	25.4	0.0–34.2

**Table 4:** Chelators use recommendations (SmPCs and Guidelines).

Age: >2 and <6 y	*SmPCs information:* the only approved drug in this group of age is DFO. Oral chelators can be used if DFO is refused, inadequate, or contraindicated. No data or few data are available in children < 6 years for oral chelators (DFP and DFX). Few data between 6 and 10 years for DFP.
	*UK guideline:* DFO is recommended as first-line treatment, DFX as second line.
	*TIF guideline:* DFO is recommended as first-line treatment, oral chelators as second line. DFP above 10 years.
	*Italian guideline: *DFO is recommended as first-line treatment. The use of oral chelators as first-line therapy should be considered investigational and should only be performed within clinical trials or registries.

Age: children >6 y and adults	*SmPCs information:* DFO and DFX approved. DFP approved if DFO is refused, inadequate, or contraindicated. DFP demonstrated to be superior to deferoxamine in decreasing cardiac iron load. (Ferriprox updated SmPC, 2010).
	*UK guideline:* DFO as first-line treatment, DFX as second line, DFP (in combination with DFO) if abnormal cardiac function (T2* or ECHO or clinical symptoms).
	*TIF guideline:* Oral chelators can be used if DFO is refused, inadequate, or contraindicated. Off label use of DFP should be avoided. For patients with very high levels of heart iron or cardiac dysfunction, 24-hour treatment with deferoxamine and daily therapy with deferiprone should be considered.
	*Italian guideline:* DFO is recommended as first-line treatment where oral chelators therapy should be considered investigational and should only be performed within clinical trials or registries.
